# Comment on “CPEO and Mitochondrial Myopathy in a Patient with *DGUOK* Compound Heterozygous Pathogenetic Variant and mtDNA Multiple Deletions”

**DOI:** 10.1155/2020/5846971

**Published:** 2020-03-07

**Authors:** Josef Finsterer

**Affiliations:** Krankenanstalt Rudolfstiftung, Vienna, Austria

With interest, we read the article by Montano et al. about a 42-year-old Italian female with ptosis, ophthalmoparesis, dysphagia, exercise intolerance, and myalgias [1]. The phenotype was attributed to the compound heterozygous mutations c.462T>A and c.707 + 2T>G in the *DGUOK* gene secondarily causing multiple mtDNA deletions [1]. We have the following comments and concerns.

Since the patient had anxiety disorder, it would be interesting to know if there were any other cerebral abnormalities clinically or on cerebral MRI. Cerebral imaging is crucial as the central nervous system (CNS) in *DGUOK*-related mitochondrial disorders (MIDs) is frequently affected and may even dominate the phenotype. Cerebral MRI may show abnormal myelination infratentorially and bilateral hyperintensity of the pallidi [2].

Dysphagia may have been due to affection of the smooth muscle cells, autonomic innervation, or due to affection of the brain. Cerebral MRI could be helpful to confirm or exclude a CNS cause of dysphagia. Autonomic testing could be helpful to investigate if there was autonomic compromise.

Though a muscle biopsy was taken, biochemical investigation of the muscle homogenate and detailed descriptions of immunostaining were not provided. Thus, it would be interesting to know which of the respiratory complexes showed reduced activity.

Since *DGUOK* mutations may also cause mtDNA depletion [3], it is worthwhile to investigate if the amount of mtDNA in a patient carrying a *DGUOK* mutation is normal or if there is mtDNA depletion. Patients with mtDNA depletion due to *DGUOK* mutations may have a worse prognosis than those without mtDNA depletion [4].

*DGUOK* mutations may not only manifest in the skeletal muscle (ptosis, CPEO, generalised myopathy, and rhabdomyolysis) and the brain (psychomotor retardation, cognitive impairment, nystagmus, Parkinsonism, and generalised hypotonia) but also in a number of other organs/tissues, such as the eyes (retinal blindness and cataract), the ears (sensorineural deafness), the liver (coagulation disorder, hypoglycemia, hemochromatosis, cholestasis, hepatomegaly, hepatocellular carcinoma, hepatopathy, portal hypertension, idiopathic hepatitis, liver failure, steatosis, and iron deposits) [5], the intestines, and the kidneys (renal failure). There may be lactic acidosis, elevated ferritin, elevated transferrin saturation, and elevated serum amino acids [6]. Thus, it is worthwhile that patients carrying *DGUOK* variants are prospectively investigated for clinical or subclinical manifestations described above.

To assess if a *DGUOK* mutation occurred sporadically or was inherited, it could be helpful that the parents and other first-degree relatives undergo a clinical neurological exam and genetic investigations.

Overall, this interesting case report could be more meaningful if biochemical investigations of the muscle or liver were presented, if cerebral MRI results were shown, if first degree relatives were investigated clinically and for the *DGUOK* variants, and if the patient was prospectively investigated for subclinical or mildly manifesting multisystem disease.

## References

[1] Montano V., Simoncini C., Calì C. L., Legati A., Siciliano G., Mancuso M. (2019). CPEO and mitochondrial myopathy in a patient with DGUOK compound heterozygous pathogenetic variant and mtDNA multiple deletions. *Case Reports in Neurological Medicine*.

[2] Brahimi N., Jambou M., Sarzi E. (2009). The first founder DGUOK mutation associated with hepatocerebral mitochondrial DNA depletion syndrome. *Molecular Genetics and Metabolism*.

[3] Copeland W. C. (2012). Defects in mitochondrial DNA replication and human disease. *Critical Reviews in Biochemistry and Molecular Biology*.

[4] Fang W., Song P., Xie X. (2017). A fatal case of mitochondrial DNA depletion syndrome with novel compound heterozygous variants in the deoxyguanosine kinase gene. *Oncotarget*.

[5] Waich S., Roscher A., Brunner-Krainz M. (2018). Severe DGUOK deficiency in Austria: a six-patient series. *Journal of Pediatric Gastroenterology and Nutrition*.

[6] Üot Ö., Hişmi B., Kılıç M. (2017). Deoxyguanosine kinase deficiency: a report of four patients. *Journal of Pediatric Endocrinology and Metabolism*.

